# Wildlife Infection of Peste des Petits Ruminants Detected in China, 2024

**DOI:** 10.3390/vetsci11100489

**Published:** 2024-10-09

**Authors:** Jiao Xu, Zebin Qu, Yingli Wang, Weijie Ren, Shan Liu, Yanli Zou, Na Su, Jingyue Bao, Zhiliang Wang

**Affiliations:** 1 China Animal Health and Epidemiology Center, Qingdao 266032, China; xujiao@cahec.cn (J.X.);; 2 College of Veterinary Medicine, Qingdao Agricultural University, Qingdao 266000, China

**Keywords:** peste des petits ruminants virus, infection, genome, evolution, phylogenetic analysis

## Abstract

**Simple Summary:**

In this study, we reported a peste des petits ruminants virus infection case in wild animals, and a virus strain was subsequently sequenced. Our results showed the existence of peste des petits ruminants virus in wild small ruminants in China, which was found to be closely related to the peste des petits ruminants virus isolated in China between 2013 and 2014. Our findings indicated that more attention should be paid to the prevention and control of wildlife diseases because there is the possibility of wild animals infecting domestic animals.

**Abstract:**

In 2013, the second outbreak of peste des petits ruminants occurred in China, leading to a spillover in more than 20 provinces and municipalities over the next few months. Thereafter, the epidemic situation was stable owing to strict prevention and control measures. In February 2024, several bharals and argali with suspected symptoms of PPR were discovered in Rutog country, Tibet Autonomous Region. Samples collected from these animals were delivered to our laboratory for diagnosis; the results of fluorescence quantitative reverse-transcription (RT) PCR indicated that all samples were positive for PPR viral RNA. The N and F gene fragments were amplified successfully via RT-PCR, and these results confirmed that these animals were infected with PPRV. A PPRV strain (subsequently named ChinaTibet2024) was sequenced, and its genome length was 15,954 nucleotides. A phylogenetic tree analysis using N and F genes and viral genomes showed that the ChinaTibet2024 genome was classified into lineage IV of the PRRV genotypes. The genome of the ChinaTibet2024 strain was found to be closely related to PPRVs isolated in China between 2013 and 2014. A base insertion and a base deletion were detected in the M gene 5′ untranslated region. Results indicated that the prevalent PPRV strains in China did not show significant changes and that special attention should be paid to the surveillance of wild animals as an important part of PPR prevention and control.

## 1. Introduction

Peste des petits ruminants (PPR) is a highly contagious infectious disease that affects small wild or domestic ruminants. PPR is caused by peste des petits ruminants virus (PPRV), belonging to the species *Morbillivirus caprinae* (family *Paramyxoviridae*, subfamily *Orthoparamyxovirinae*), which has a single-stranded negative-sense RNA genome. The incubation period of PPR is typically 4–6 days but might range between 3 and 10 days. In animals infected with PPRV, the symptoms are usually severe pyrexia, mucopurulent nasal and ocular discharges, cough dyspnea, necrotic stomatitis, and diarrhea. The mortality of PPR can be as high as 100%. Therefore, PPR is on the list of notifiable diseases, and member countries and regions are obliged to report cases to the World Organization of Animal Health (WOAH) [1]. Currently, approximately 62.5% of the small ruminant population worldwide are at risk of being infected by PPRV, which severely impairs livestock breeding and production, especially in developing countries and regions [2]. According to the FAO and WOAH, PPR has been identified as a target for global control and eradication by 2030.

PPR was first reported in Cote d’Ivoire in 1942 [3] and is presently circulating in Africa and Asia; it was also recently identified in some European countries [4,5]. To date, only one serotype of PPRV has been detected, which is further divided into lineages I–IV on the basis of fusion proteins and nucleoproteins [6]. In 2007, China reported PPR for the first time in Tibet; it was quickly eliminated through the slaughter of infected animals, quarantine, and vaccination. However, in 2013, PPR re-emerged in the Xinjiang Uygur Autonomous Region and has rapidly spilled over to several provinces in China since then [7,8]. Therefore, a series of measures were adopted to control PPR transmission, including culling, restrictions on the transportation of live sheep and goats across provinces, and mandatory vaccination in areas with high epidemic risk, which consequently prevented a severe outbreak once again. To date, a national epidemiological investigation into PPR is conducted annually in all provinces, autonomous regions, and municipalities of China (except in Hong Kong, Macao, and Taiwan).

Similar to domestic ruminants, wild ruminants are also susceptible to PPRV and are commonly considered sentinels indicating the spillover of PPRV from domestic animals [9]. Typically, the wild species have higher morbidity and mortality as a result of PPR because some wild species are naturally more susceptible to the clinical or pathological effects of the virus than various breeds of domestic sheep and goats. Various wild ruminant species have been reported to be infected with PPRV, including the gemsbok (*Oryx gazelle*) [10], goitered gazelle (*Gazella subgutturosa*) [11], bharal (*Pseudois nayaur*) [8], alpine ibex (*Capra ibex*) [12], and argali (*Ovis ammon*) [12]. In areas where wildlife is widely distributed, especially in the western provinces of China, the frequency of contact between wildlife and domestic ruminants is high, increasing the likelihood of PPRV transmission from infected domestic animals to wild ruminants, and the long-distance migration of wildlife further expands the range of PPRV transmission.

Bao et al. confirmed that the virus isolated in 2007 and 2013 in China belonged to different clades, suggesting that the source of the two PPR infections was different. The genome length of strains isolated in 2013–2014 in China was 15,954 nt, with a 6 nt insert (TCCCTC) in the 5′ untranslated region (UTR) of the F gene, which was unique in comparison with all other strains [13]. Recent studies indicate that almost all isolates from infected ruminants (wild and domestic) in China shared the same genotype (lineage IV) [12,14,15]. Notably, Zhou et al. reported that the PPRV strain isolated from water deer *(Rusa unicolor)* was classified into lineage II. Zhou et al. constructed a phylogenetic tree of N gene nucleotide sequences using the neighbor-joining method based on a Tamura three-parameter model and bootstrap analysis. The analysis placed their N gene sequence in lineage II, most closely related to Nigeria/75/1 [16].

In 2024, several dying and dead bharals (*Pseudois nayaur*) and argali *(Ovis ammon)* with clinical signs consistent with PPRV infection were reported in the southern parts of Rutog County, Tibet Autonomous Region. Nucleic acid-based diagnostic tests performed in our laboratory (WOAH PPR Reference Laboratory) indicated that all reported animals were infected with PPRV. These cases of infection in wildlife were subsequently reported to WOAH.

To investigate to the evolution of PPRV in China, a detailed epidemiological analysis and viral sequencing was performed in this study.

## 2. Materials and Methods

### 2.1. Peste des Petits Ruminants Epidemic in China in 2024

In February 2024, the PPR infection of wildlife was reported in the southern parts of Rutog County, Tibet Autonomous Region, including 65 bharals and argali; 64 of these were dead and one bharal was killed and disposed of.

### 2.2. Clinical Symptoms of Diseased Animals

All animals with suspected PPRV infection showed similar clinical signs before death, including conjunctivitis, oral mucosal congestion and necrosis, breathing difficulties, pyrexia, and diarrhea. The autopsies of dead bharals and argali revealed bronchoalveolitis and intestinal mucosal hemorrhage.

### 2.3. RNA Extraction and Quantitative Reverse-Transcription PCR

All samples from 65 animals were collected and stored at −80 °C for diagnosis using real-time PCR. All samples were thawed and processed in a biosafety level III laboratory. RNA extraction was performed using a magnetic bead-based viral DNA/RNA extraction kit (Tianlong, China) according to manufacturer’s instructions. Reaction buffer was prepared including plate, primers, probe, and polymerase buffer. The primers and probe used for fluorescence quantitative reverse-transcription (RT) PCR are shown in Table S1. The following program of the ABI QuantStudio 7 system was used: reverse transcription at 50 °C for 15 min and pre-denaturation at 95 °C for 5 min, followed by 40 cycles of denaturation at 95 °C for 15 s and annealing at 60 °C for 30 s. Fluorescence signals were collected at 59 °C in each cycle. Samples with cycle threshold (Ct) values of ≤38 and >38 were considered positive and negative, respectively.

### 2.4. Reverse-Transcription PCR

N and F gene fragments and viral genome were amplified using RT-PCR using the following program: reverse transcription at 50 °C for 15 min; pre-denaturation at 95 °C for 5 min; 40 cycles of 95 °C for 25 s, 56 °C for 30 s, and 72 °C for 45 s; followed by extension for 5 min at 72 °C. Two pairs of primers were designed to target the N and F genes, respectively, and nine pairs of primers were designed to target the whole genome of PPRV (Tables S2 and S3, respectively). The N75/1 vaccine strain and DEPC-treated water were used as positive and negative controls, respectively.

### 2.5. Genomic Sequencing

RT-PCR amplification products were separated by 1.5% agarose gel electrophoresis and purified using a DNA gel extraction kit. The amplicons were sequenced by Sangon Biotech (Shanghai, China) using standard Sanger methods. The corresponding sequencing results were spliced using SeqMan software (version 7.1.0).

### 2.6. Genomic Alignment and Phylogenetic Analysis

Reference sequences of all lineages of PPRV were obtained from NCBI, and sequence alignment was performed using ClustalW in Mega X. The phylogenetic analysis of the N gene, F gene, and whole genome of the newly sequenced strains and the reference sequences was performed using the neighbor joining method in Mega X; a bootstrap value of 1000 pseudoreplicates was applied. The reference strains used in this study are shown in Supplementary Materials (Table S4).

## 3. Results

### 3.1. Nucleic Acid Analysis for Peste des Petits Ruminants Virus

**Figure 1:**
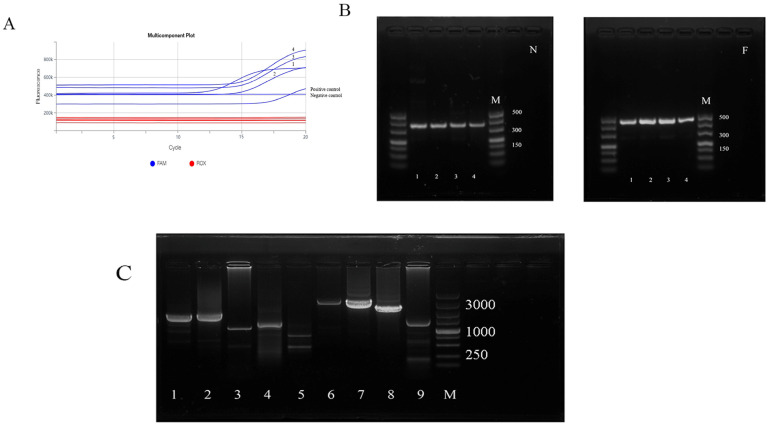
Laboratory diagnosis of the infection (**A**) fluorescence quantitative reverse-transcription PCR, (**B**) the amplification of N and F genes, and (**C**) the amplification of peste des petits ruminants virus (PPRV) genome.

All samples collected from animals and positive control showed a distinctive amplification curve. Four samples collected from animals that showed classical PPR symptoms were strongly positive for PPRV nucleic acid (Ct value ranged from 10 to 15) (Figure 1A); these four samples were selected for further molecular characterization. No specific amplification curve was observed in the negative control.

### 3.2. Amplification of N and F Genes and Genomic Fragments

As shown in Figure 1B, the partial amplification of both N and F genes was achieved in all samples, with amplification products of 351 bp and 448 bp, respectively. Genome fragment amplification results are shown in Figure 1C. The PPRV genome was divided into nine segments (numbered 1–9), all of which were amplified, with the correct product size.

### 3.3. Phylogenetic Analysis and Sequence Alignment

**Figure 2:**
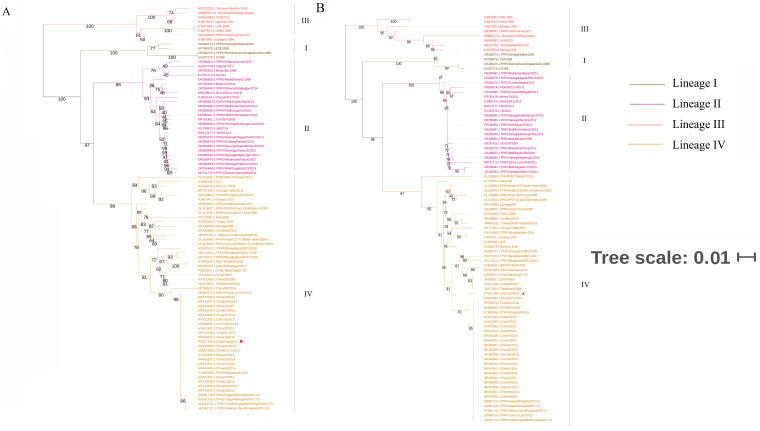
Phylogenetic analysis based on peste des petits ruminants virus (PPRV): N gene (**A**); F gene (**B**). Viral strain studied in this research was marked using triangles.

The consensus phylogenetic trees of PPRV based on N gene sequences and F gene sequences (Figure 2A and Figure 2B, respectively) indicated that the PPRV strain isolated in 2024 (ChinaTibet2024) belonged to lineage IV and shared high genetic similarity with the strains isolated in China (2013–2014) and Mongolia (2016–2017) but low genetic similarity with strains isolated in Tibet, China (2007–2008), and neighboring countries (India and Bangladesh) in recent years.

**Figure 3:**
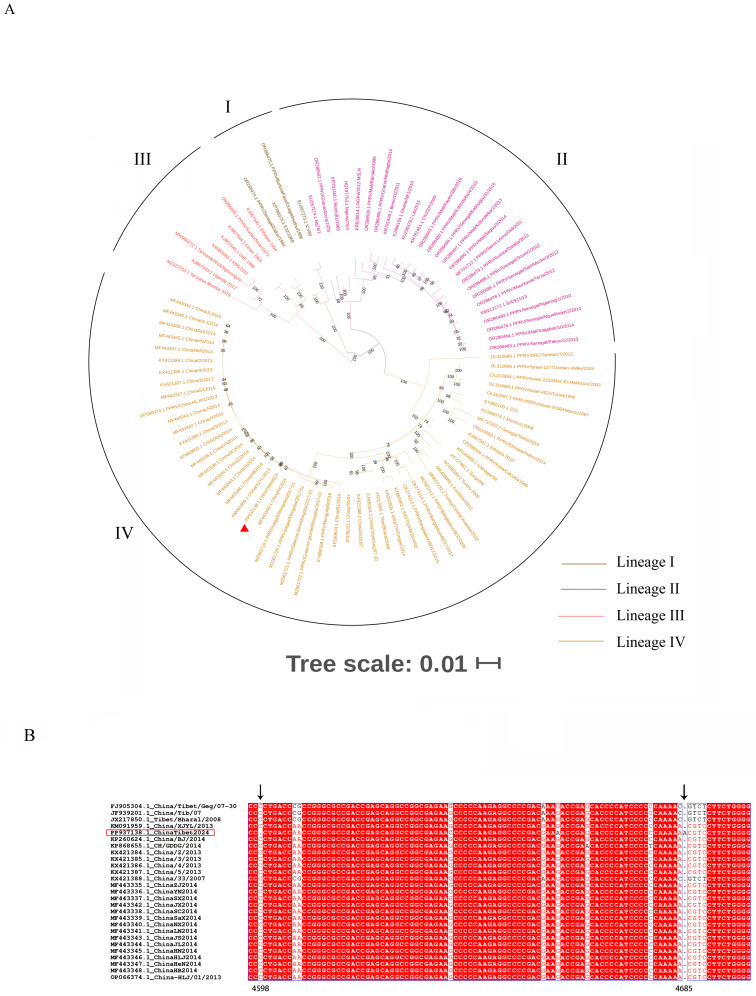
Phylogenetic analysis based on peste des petits ruminants virus (PPRV) full genome, viral strain studied in this research was marked using triangles. (**A**) and sequence alignment results of partial M gene 5′ untranslated region (4596–4698) between the ChinaTibet2024 strain and other strains isolated in China (**B**).

Similarly, the phylogenetic tree based on the PPRV genome (Figure 3A) indicated that the ChinaTibet2024 strain belonged to lineage IV. All PPRV genomes from China were grouped into two clades: the first clade included the strains isolated in 2007–2008, and the other clade included the strains isolated in 2013–2014. The strains ChinaTibet2024 and China/XJYL/2013 were clustered in the same sub-branch, indicating a close genetic relationship and high homology (99%; Table 1). The results of sequence alignment between the ChinaTibet2024 strain and strains isolated in China are shown in Figure 3B, a base insertion and a base deletion were detected in the M gene 5′ untranslated region. Compared with the strains isolated in Tibet in 2007, the ChinaTibet2024 strain genome (15,954 bp in length) showed a 6 nt insert in the 5′ UTR of the F gene—a feature consistent with strains isolated in 2013–2014 in China [13]; moreover, a base deletion and base insertion were observed at positions 4598 and 4695, respectively.

### 3.4. Nucleotide and Amino Acid Diversity between ChinaTibet2024 and China/XJYL/2013

The results of nucleotide and amino acid diversity between ChinaTibet2024 and China/XJYL/2013 are shown in Table 1. The nucleotide diversity between the two strains was 1%; in total, 160 nucleotide differences were identified between two strains, with 62 nucleotide substitutions in noncoding regions. Among noncoding regions, the highest nucleotide diversity between two strains was observed in the 5′ UTR of the F gene (5.15%), followed by 3′ UTR of the F gene (4.22%) and 5′ UTR of the M gene (4.06%). Among coding sequence (CDS) regions, the highest nucleotide and amino acid diversity were observed in the H gene CDS (0.98%) and P gene CDS (1.38%), respectively. No nucleotide variable sites were observed in the 3′ UTR of the M, H, and L genes. Amino acid substitution analysis of ChinaTibet2024 in comparison with China/XJYL/2013 showed that the most frequently observed amino acid substitutions were serine to asparagine (S to N) and arginine to lysine (R to K).

## 4. Discussion

The present study reports the infection of PPR in wild animals in China. In general, the number of PPR infections associated with wildlife is considerably lower than in domestic ruminants. However, to investigate to the evolution of PPRV in China and develop relevant control measures, a detailed epidemiological analysis and viral sequencing was performed in this study. Owing to its biological risk and threat to small domestic ruminants, PPR has been classified as a list A disease, and urgent and strict measures need to be implemented in China for PPR prevention and the control or mandatory extermination of infected animals. In the present study, all diseased animals showed symptoms consistent with PPR, which raised serious concerns. A new PPR infection case was confirmed via PCR detection based on PPRV N and F genes. Previous research suggests that PPRV spillover from infected domestic ruminants into wild animals leads to infection [9,10]. Considering the fragile character of PPRV, transmission by fomites is unlikely [17]; PPRV transmission primarily occurs through the respiratory route, i.e., through exposure to short-range aerosols produced by sneezing and coughing. The Tibetan plateau is rich in wildlife resources, and a cage-free model of livestock production is common in this area. Therefore, we speculate that wild ruminants are infected through direct contact with domestic animals in a setting where domestic and wild ruminants share pastures.

PPRV is widely distributed in Africa and Asia, with lineages I and II mostly prevalent in west Africa; lineage III in east Africa; and lineage IV in the Arabian Peninsula, Middle East, and Southern and Eastern Asia [18]. Phylogenetic analysis based on the N gene, F gene, and whole-genome sequence indicated that the newly isolated PPRV strain belonged to lineage IV, which is consistent with previous findings in China and neighboring country Mongolia [13,19]. Notably, an isolated study has reported that PPRV belonging to lineage II was detected in China [16]; however, the possibility cannot be excluded that the detected strain was Nigeria 75/1 (a vaccine strain) because of the mandatory vaccination in some areas [20]. Phylogenetic analysis also showed a close genetic relationship between the PPRV strain isolated in 2024 and those isolated in 2013–2014, suggesting that a similar PPRV strain was associated with these cases and not a novel PPRV strain.

The PPRV genome encodes six structural proteins—including the nucleocapsid protein (N), matrix protein (M), phosphoprotein (P), fusion protein (F), hemagglutinin (H), and polymerase (L)—and two nonstructural proteins—C and V—in the order 3′-N-P/C/V-M-F-H-L-5′ [21]; the transcriptional units are separated from each other by conserved intergenic trinucleotides [22]. In the present study, owing to the closest genetic relationship between ChinaTibet2024 and China/XJYL/2013, the nucleotide and amino acid diversity analysis were estimated. The highest amino acid diversity was observed in P gene CDS. Previous studies report that the P protein is involved in viral genome transcription and replication [23,24]. Moreover, to our knowledge, this is the first study to report base insertion and deletion in the 5′ UTR of the M gene; it needs to be further investigated whether these mutations affect the characteristics of PPRV (especially virulence and contagiousness).

The role of wildlife in the spread of PPRV is not completely understood; however, it is regarded as a weak link in PPR epidemic control. Almost all wildlife is out of human control, and wildlife vaccination is challenging, which slows the development of an immunological barrier to PPRV. Furthermore, the long-distance migration of infected wildlife increases the risk of viral transmission, posing a significant threat to domestic ruminants, especially in areas with uninfected wildlife and without mandatory vaccination. Surveillance of wildlife should be reinforced to detect PPRV at the early stage so that measures can be taken to interrupt viral transmission. Moreover, strain isolation from wildlife should be performed, which is fundamental to understanding the genetic nature of theses strains; this knowledge would also be helpful to fill the epidemiological gaps regarding wild small ruminants.

Thus, in the present study, we report the occurrence of PPR infection in wildlife in China. The viral phylogenetic analysis suggested that the PPRV strains in China continue to evolve although they share high similarity with the strains isolated in China in 2013–2014. Our findings indicated that the virus exists in wild animals, although the source of the virus that in this case has not been ascertained, which reminds us that special attention should be paid to the surveillance of wild animals as an important part of PPR prevention and control, and the significant potential threat of PPR to large domestic and wild animal populations warrants further research and necessitates the continued surveillance of PPR.

## Figures and Tables

**Table 1:** Sequences and amino acid diversity compared with China/XJYL/2013.

Region	Length (bp)	Nucleotide Variable Sites	Diversity (%)	Encoding Amino Acid	Amino Acid Variable Sites	Diversity (%)	Amino Acid Position	Amino Acid Mutation
Leader	52	2	3.85	/	/	/	/	/
N 3′ UTR	52	2	3.85	/	/	/	/	/
N cds	1578	15	0.95	525	2	0.38	442	I-T
							515	S-N
N 5′ UTR	59	2	3.39	/	/	/	/	/
P 3′ UTR	59	1	1.69	/	/	/	/	/
P cds	1530	10	0.65	509	7	1.38	30	R-K
							93	A-T
							140	V-I
							174	N-D
							248	I-T
							266	N-S
							339	I-L
P 5′ UTR	66	1	1.52	/	/	/	/	/
M 3′ UTR	32	0	0.00	/	/	/	/	/
M cds	1008	5	0.50	335	0	0	/	/
M 5′ UTR	443	18	4.06	/	/	/	/	/
F 3′ UTR	640	27	4.22	/	/	/	/	/
F cds	1641	10	0.61	546	1	0.18	401	S-N
F 5′ UTR	136	7	5.15	/	/	/	/	/
H 3′ UTR	20	0	0.00	/	/	/	/	/
H cds	1830	18	0.98	609	5	0.82	27	R-K
							309	S-N
							438	P-L
							488	R-K
							609	V-A
H 5′ UTR	107	2	1.87	/	/	/	/	/
L 3′ UTR	22	0	0.00	/	/	/	/	/
L cds	6552	40	0.61	2183	8	0.37	78	S-G
							122	H-R
							138	G-S
							843	V-I
							1122	G-A
							1621	S-G
							2114	P-S
							2162	T-A
L 5′ UTR	69	0	0.00	/	/	/	/	/
Trailer	37	0	0.00	/	/	/	/	/
Total	15,933	160	1.00	4707	23	0.49	/	/

## Data Availability

The original contributions presented in the study are included in the article/Supplementary Materials; further inquiries can be directed to the corresponding author. The genome sequence data generated in this research have been deposited in the GenBank database under accession number PP937138.

## Supplementary Materials

The following supporting information can be downloaded at: https://www.mdpi.com/article/10.3390/vetsci11100489/s1, Table S1: Primers and probe used for PPRV fluorescence quantitative PCR detection; Table S2: Primers used for PPRV genome amplification; Table S3: Primers used for the amplification of PPRV N and F fragments; Table S4: Details of reference strains used in this study.
